# Superficial mucoceles in cancer patients: a retrospective series from a Stomatology unit

**DOI:** 10.4317/medoral.25972

**Published:** 2023-07-10

**Authors:** Daniele Heguedusch, Saygo Tomo, Oslei Paes de Almeida, Fabio A Alves

**Affiliations:** 1 DDS, Resident. Department of Stomatology, A.C. Camargo Cancer Center, Sao Paulo, Brazil; 2 DDS, Ph.D. candidate, Department of Stomatology, University of Sao Paulo School of Dentistry, Sao Paulo, Brazil; 3 DDS, MSc, Ph.D., Resident. Department of Stomatology, A.C. Camargo Cancer Center, Sao Paulo, Brazil; 4 DDS, MSc, Ph.D., Full Professor. Department of Oral Pathology, School of Dentistry, Unicamp, Piracicaba, Brazil; 5 DDS, MSc, Ph.D., Head of Department. Department of Stomatology, A.C. Camargo Cancer Center, Sao Paulo, Brazil; 6 DDS, MSc, Ph.D., Associate Professor. Department of Stomatology, University of Sao Paulo School of Dentistry, Sao Paulo, Brazil

## Abstract

**Background:**

The aim of this study is to relate all the superficial mucoceles found in a cancer center, described the association with oncological conditions, and discuss its etiology and pathology that we found in the past few years.

**Material and Methods:**

Sixteen cases of superficial mucocele were retrieved from the patients’ records of the Stomatology Department of the A. C. Camargo Cancer Center, São Paulo, Brazil, and demographic and clinical data were collected from electronic medical records.

**Results:**

There were 16 patients, 8 patients were men and 8 women, with ages varying from 26 to 70 years old. Superficial mucoceles were observed in patients submitted to head and neck radiotherapy (*n*=6), graft versus host disease (*n*=4), one associated with oral mucositis related to allogenic bone marrow stem cells transplantation (*n*=1), systemic lupus (*n*=1), Sjögren’s syndrome (*n*=1), oral lichenoid lesion associated with pembrolizumab (*n*=1) and no local or systemic inflammatory associated found (*n*=2).

**Conclusions:**

This study reports a series of superficial mucoceles from a single stomatology unit. Most patients had superficial mucoceles secondary to head and neck radiotherapy and graft versus host diseases. However, two patients (12.5%) had mucoceles related to systemic inflammatory conditions (Sjögren’s Syndrome and Systemic Lupus).

** Key words:**Superficial mucocele, oncological treatment, oral lesions.

## Introduction

Mucocele is a common oral mucosal lesion resulting primarily from trauma to the minor salivary glands, that may develop at virtually any location where minor salivary glands occur, but the lower labial mucosa is more affected (1). Clinically, the mucocele is characterized by a discrete nonpainful dome-shaped swelling of the mucosa (1). No sex predilection is observed, but it is more frequent in children, adolescents, and young adults (1).

Superficial mucoceles (SM) was first described in 1988 (2), as a small, translucent, tense, single or multiple subepithelial vesicles affecting the oral mucosa. Occasionally the lesions are persistently recurrent, causing slight discomfort to the patient. Interestingly recently, SM has been described in association with other systemic conditions such as lichen planus (3), chronic graft-versus-host disease (GVHD) (4,5), lichenoid disorders (6), and after head and neck radiotherapy (HNRT) (7,8).

Different from conventional mucoceles, SM can be associated with local or systemic diseases. Moreover, clinicians should be alert about the appearance of SM and its relationship with other diseases. Hereby we describe a series of patients with SM from a single Stomatology unit at a Cancer Center.

## Material and Methods

This is a cross-sectional retrospective study conducted in the Stomatology Department at A. C. Camargo Cancer Center, São Paulo, Brazil, between January 2015 and June 2022. This study was approved by Institutional Committee on Ethics of A. C. Camargo Cancer Center under the nº4.478.095.

A total of 48 cases of patients who had a confirmed oral mucocele diagnosis were evaluated. The clinical aspects of conventional or superficial mucoceles were also collected from the patient’s photography file and confirmed cases of oral SM were selected.

- Inclusion Criteria

Patients presenting oral superficial mucoceles.

- Exclusion Criteria

Other kinds of oral mucoceles (conventional).

Demographic data including age, gender, and oncologic history were collected from each patient. Records of clinical notes were collected, including medications, complaints, site of lesion, photographs, concurrent chemotherapy, radiotherapy, date of transplant if applicable, and immunotherapy. A descriptive analysis is provided.

## Results

Of a total of 48 patients, 16 presented SM (33.3%). Considering only the patient’s first visit, multiple SM were observed in 6 patients and a single lesion in the other 10 patients. Of the 16 patients, 15 had only one region of the mouth affected (the soft palate was the main site involved) and one patient had simultaneous lesions on the soft palate and lip. Regarding sex, 8 patients were men and 8 women, with ages varying from 26 to 70 years old (Table 1).

**Table 1:**
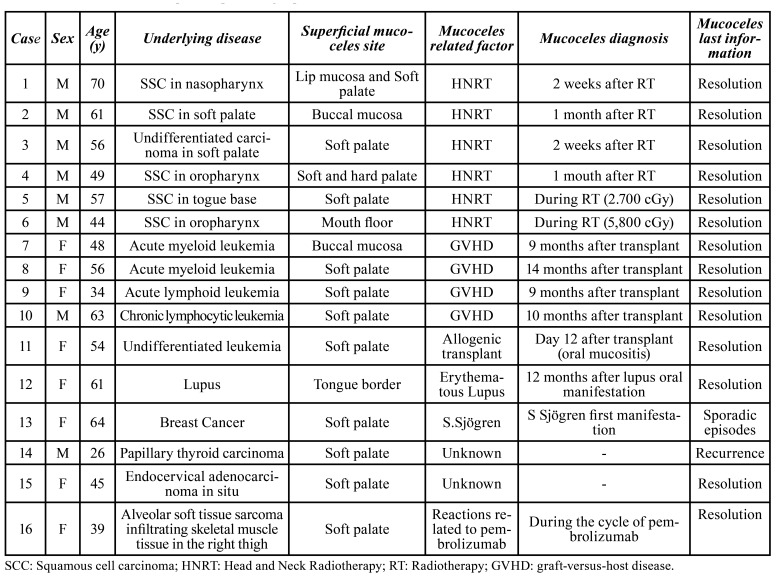
Clinical features of 15 patients presenting superficial mucoceles.

**Figure 1:**
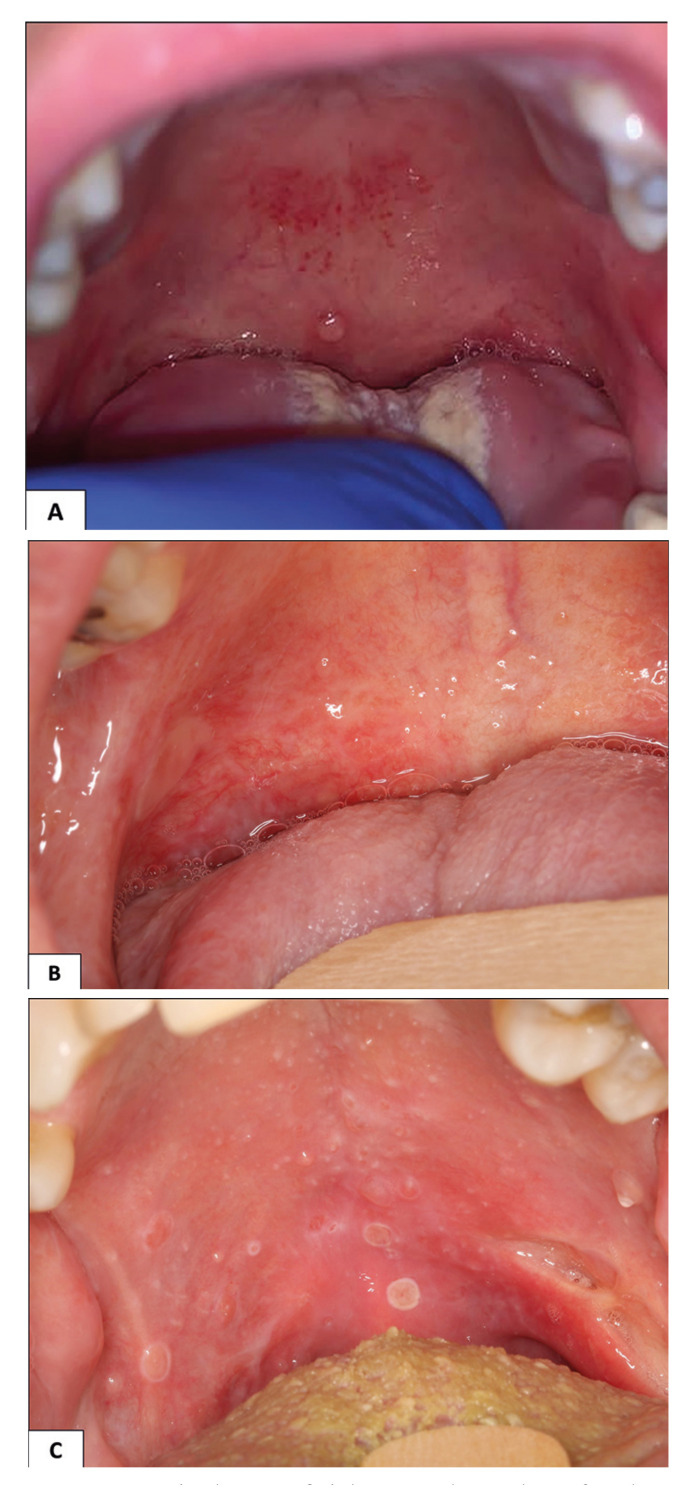
A: a single superficial mucocele on the soft palate measuring 2mm, in a patient who received during allogenic transplant of undifferentiated leukemia B:multiples superficial mucoceles associated to lichenoid reaction by immune-related adverse events. C: multiples superficial mucocele in on the soft palate measuring 2-3mm in an irradiated patient (1 month after radiotherapy).

**Figure 2:**
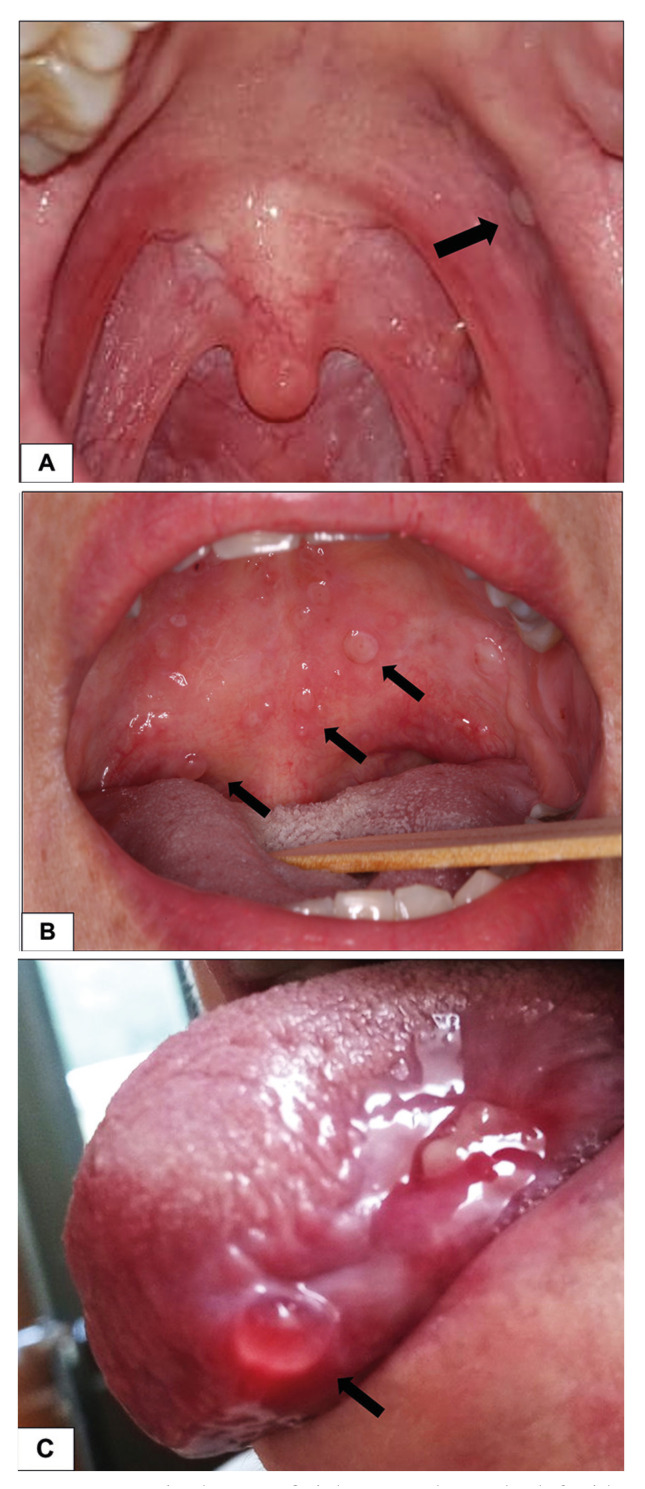
A: a single superficial mucocele on the left side of the soft palate in a 26-years old man without local or systemic disease. B: multiples superficial mucocele in on the soft palate measuring 2 - 4mm in a patient with Sjögren’s Syndrome. C: a single superficial mucocele measuring 7mm in on the border of the tongue of a patient with an erythematous lupus patient.

SM was observed in patients submitted to HNRT (*n*=6), GVHD (*n*=4), and one each with oral mucositis related to allogenic bone marrow stem cells transplantation, systemic lupus, Sjögren’s syndrome (SS) and oral lichenoid lesion associated to pembrolizumab (Fig. 1, Fig. 2). However, no associated factors were identified in the two patients. One of them was a 25-year-old man who had thyroid cancer treated with surgery one year previously. In his first appointment, multiple small vesicles were observed on the soft palate, that appeared and disappeared sporadically (Fig. 2). The other patient was a 45-year-old woman submitted to surgery due to an in situ endocervical adenocarcinoma, who in the first visit, presented with a 1-week duration single vesicle on soft palate. With a clinical diagnosis of SM, this lesion was submitted to excision due to local discomfort. For both patients, no local or systemic inflammatory or other conditions were identified (Table 1).

In 14 out of 16 cases, the diagnosis was performed by clinical evaluation alone. In cases 13 (SS) and 15 (unknown factor) (Table 1) excisional biopsies were also performed. Microscopically, case 13 showed an empty space adjacent to the superficial epithelium (corresponding to mucous extravasation) and chronic inflammatory infiltrate. In case 15 a small nodule (3mm) was biopsied, but only minor salivary glands associated with chronic inflammatory cells were found.

No specific treatment was performed on all these cases; however topical clobetasol propionate 0.05% was prescribed to 6 patients twice a day (3 patients with GVHD, and one each with radio-induced mucositis, erythematous lupus and oral lichenoid lesion associated to pembrolizumab) to control local inflammation related to underlying diseases, and all these cases had complete resolution of the SMs, without recurrence. Spontaneous regression was observed in other 7 patients (5 submitted to HNRT, 1 after oral mucositis during bone marrow transplantation, and 1 patient who had GVHD. Two patients (13 and 14 patients) continue to present the SMs and another patient (patient 15) who was treated by excisional biopsy did not present any lesions (Table 1).

## Discussion

The first description of SM in the literature, made by Eveson, dates to 1988, as small, translucent, subepithelial vesicles affecting the oral mucosa (2). Although SM may occur in healthy patients, recent evidence suggests that this lesion is more frequently associated to underlying primary conditions. It is well-described the association of SM with inflammatory oral conditions such as lichen planus, lichenoid lesions, allergic stomatitis, toothpaste sensitivity, GVHD (3,4,9-13), and HNRT (8,14). Its etiopathogenesis is not completely known. Jensen suggested that mucous plugs in the intraepithelial-cell-lined portions of the minor salivary glands ducts could increase pressure and result in duct rupture and consequently the development of SM (15). On the other hand, an alternative hypothesis suggests an inflammatory mechanism. Lymphocytic infiltrate can obstruct the accessory gland duct, leading to duct rupture and subsequent extravasation of the subepithelial mucosa (4). Therefore, it is possible that the direct damage caused by HNRDT and chronic inflammation of the mucosa may contribute to this pathologic process.

In this series, 12 of 16 patients had lesions associated with oncological treatment. Six patients developed lesions related to HNRT, which was performed to treat oropharynx and nasopharynx cancers. In two patients the lesions appeared during the HNRT and in four 2 weeks up to 1 month after the radiotherapy (Fig. 1). Such SMs are possibly due to the high dose of RT delivered on the oral mucosa affecting directly the minor salivary glands but may also be due to local inflammation associated with mucositis (Fig. 1). The latter possibility could be a phenomenon like that of other oral inflammatory conditions described above. Irradiation-related SM was first related by Keshet *et al*. who shows two cases of multiples SM after HNRT (14). Similarly, Prado-Ribeiro *et al*. reported 10 cases of SMs in patients receiving HNRT (8). In both studies, the patients did not have any complaints, and patients were only followed without treatment, with spontaneous resolution of the lesions.

Treister *et al*. evaluated 331 patients with GVHD and SMs were presented in 141 patients, evidencing an important correlation between the two diseases (5). Similarly, 4 out of 16 patients in this study had GVHD. Three patients presented SM located in the soft palate and one in the buccal mucosa. Topical clobetasol was indicated to GVHD control. During the follow-up, such patients had a reduction of the oral mucosa inflammation and resolution of SM.

SM was the first complaint of the patient with SS. On her first visit, an intraoral examination revealed multiple vesicles mainly on the soft palate, which ruptured easily resulting in small painful ulcers (Fig. 2). After 6 months, the patient reported knee joint pain and SS was suspected. Schirmer’s test showed severe ocular dryness and serological tests were positive for anti-SSA confirming the diagnosis of SS. The patient was referred to rheumatologic management and she is under chloroquine use. SMs still occur sporadically after 2 years-follow-up. Salivary gland dysfunction causing xerostomia is an important feature of SS, but superficial and persistent multiple mucoceles, to the best of our knowledge, is the first time reported. Mucoceles associated with SS were reported by Katayama I *et al*. who reported two women with primary SS, both patients developed a single giant mucocele on the floor of the mouth (16). Similarly, Pinheiro JB *et al*. described a 37 years old woman presenting a single mucocele on the floor of the mouth 12 months after SS initial diagnosis (17). It is important to highlight that none of such cases were considered SM. Bermejo *et al*. reported one case of SM in a patient who already had SS and lichen planus associated with SM, and the development of SM in that case was more associated with the inflammation which cause the outlet of a small salivary duct might become blocked or rupture, causing mucus to collect below the epithelium (9).

One of our cases affected a patient receiving Pembrolizumab. Recently, immunotherapy has been used to treat various types of cancer, eventually causing oral side effects (18), emphasizing the association between immune checkpoint inhibitors and lichen planus-like reactions, bullous pemphigoid, mucous membrane pemphigoid, erythema multiforme, Stevens-Johnson syndrome, toxic epidermal necrolysis, and SS development. Our patient presented ulcers, erythema, white striae, and a single SM in the soft palate during the treatment with pembrolizumab for alveolar soft tissue sarcoma (Fig. 1). Previously, also reported a single palate SM in a patient using pembrolizumab. In both cases, the SMs emerged in inflammatory sites which are associated with oral reactions to immune checkpoint inhibitors (immune-related adverse events) (19).

## Conclusions

In summary, this study reported the relationship between SM and local and systemic inflammatory conditions. Such lesions were most often observed shortly after the end of radiotherapy and in patients presenting chronic GVHD. Moreover, a possible association between SM and two systemic inflammatory conditions (Sjögren’s Syndrome and Systemic Lupus) was also found.
